# Isolation and characterization of chitosan from Ugandan edible mushrooms, Nile perch scales and banana weevils for biomedical applications

**DOI:** 10.1038/s41598-021-81880-7

**Published:** 2021-02-18

**Authors:** Kenneth Ssekatawa, Denis K. Byarugaba, Eddie M. Wampande, Tlou N. Moja, Edward Nxumalo, Malik Maaza, Juliet Sackey, Francis Ejobi, John Baptist Kirabira

**Affiliations:** 1 College of Veterinary Medicine Animal Resources and Biosecurity, Makerere University, P. O. Box 7062, Kampala, Uganda; 2 African Center of Excellence in Materials, Product Development and Nanotechnology, College of Engineering, Design, Art and Technology, Makerere University, P. O. Box 7062, Kampala, Uganda; 3 Department of Biochemistry Faculty of Biomedical Science, Kampala International University-Western Campus, P. O. Box 71, Bushenyi, Uganda; 4 University of South Africa-Florida, Campus Private Bag X6, Florida, 1710 South Africa; 5 Nanosciences African Network (NANOAFNET), iThemba LABS-National Research Foundation, Old Faure Road, Somerset West, 7129 South Africa; 6 UNESCO-UNISA Africa Chair in Nanosciences/Nanotechnology, College of Graduate Studies, University of South Africa (UNISA), Muckleneuk Ridge, PO Box 392, Pretoria, South Africa

**Keywords:** Biophysics, Immunology, Medical research, Chemistry, Materials science

## Abstract

Of recent, immense attention has been given to chitosan in the biomedical field due to its valuable biochemical and physiological properties. Traditionally, the chief source of chitosan is chitin from crab and shrimp shells. Chitin is also an important component of fish scales, insects and fungal cell walls. Thus, the aim of this study was to isolate and characterize chitosan from locally available material for potential use in the biomedical field. Chitosan ash and nitrogen contents ranged from 1.55 to 3.5% and 6.6 to 7.0% respectively. Molecular weight varied from 291 to 348KDa. FTIR spectra revealed high degree of similarity between locally isolated chitosan and commercial chitosan with DD ranging from 77.8 to 79.1%. XRD patterns exhibited peaks at 2θ values of 19.5° for both mushroom and banana weevil chitosan while Nile perch scales chitosan registered 3 peaks at 2θ angles of 12.3°, 20.1° and 21.3° comparable to the established commercial chitosan XRD pattern. Locally isolated chitosan exhibited antimicrobial activity at a very high concentration. Ash content, moisture content, DD, FTIR spectra and XRD patterns revealed that chitosan isolated from locally available materials has physiochemical properties comparable to conventional chitosan and therefore it can be used in the biomedical field.

## Introduction

Chitin the raw material for synthesis of chitosan, is extensively abundant in nature. Like cellulose, this polymer is linear and non-polar with very low chemical reactivity but highly soluble in concentrated acids and a few flouroalcohols^1–3^. Chitin is somewhat analogous to cellulose being based on glucose repeat units; however, each glucose molecule in chitin has an acetamido group at position C-2 and found in the exoskeletons of arthropods such as insects, crustaceans, arachnids and myriapods; cell walls of fungi and possibly scales of fish providing tensile strength^1^. Due to its insolubility at physiological conditions, chitin can chemically be modified to its soluble alternatives. Chitosan the commonest derivative of chitin is mainly derived through non-enzymatic *N* deacetylation. This is achieved through cleaving off the acetyl residue (R-NHCOCH_3_) mediated by strong alkali at high temperatures. Furthermore, chitosan can be synthesized through enzymatic processes. However, owing to the high cost of deacetylases and their low chitosan productivity, enzymatic mediated chitin deacetylation is unpopular^4,5^.

Of recent, a lot of attention has been given to chitosan in the biomedical field due to its valuable biochemical and physiological properties such as biodegradability, biocompatibility, non-immunogenicity, reactivity, solubility and non-toxicity. For instance, chitosan has exhibited distinguished bioactivity not limited to only antimicrobial activity but also promotion of wound healing and immune system augmentation^6,7^. The beneficial biological properties of chitosan can be enhanced when converted to its nano scale form. Chitosan is transformed to its nanoparticles using several methods such as ionic gelation, reverse micellization, microemulsion and polyelectrolyte complexation^8,9^ Several studies have revealed that chitosan and chitosan derivatives have enhanced antimicrobial activity and are good vehicles of drugs and vaccines to the target body parts^8–11^.

Conventionally, the chief sources of chitin are crab and shrimp shells obtained as waste products in the seafood industry^12,13^. Chitin is also an important component of fish scales, arthropod exoskeleton and cell walls of fungal cells; therefore, Uganda’s edible mushrooms, banana weevils and Nile perch scales can be alternative sources of chitin and its chitosan derivatives. Production of chitin and its derivatives from renewable resources such as fishery wastes, arthropods (banana weevils) and fungi present sustainability for the ever-increasing demands of this polymer. Exploring the use of Nile perch scales and banana weevils as sources of chitosan for biomedical application will alleviate on the burden they put on the respective industries as Nile perch scales are a major waste of the fisheries industry without any application and require extra resources for proper disposal while the banana weevil is the chief banana production restraint in Uganda^14,15^. Furthermore, isolation of chitosan from mushrooms constitutes value addition.

Furthermore, antibiotic resistant bacteria are escalating in prevalence globally with consequential infections which are hard and costly to treat^16^. This is supported by emergence of carbapenem resistance in *Enterobacteriaceae* yet carbapenems such as imipenem, ertapenem, meropenem, and doripenem are the newest synthesized beta-lactam antibiotics with the broadest spectrum of activity and consequently considered the first line therapy antibiotics in the treatment of multi resistant (MDR) gram-negative pathogens^17,18^.

Drug delivery systems with antimicrobial activity such as chitosan will not only shield antibiotics from the bacterial hydrolytic enzymes but also re-potentiate the antibiotic by conferring the drug delivery system-antibiotics complex synergistic bactericidal effect^19^. Furthermore, drug delivery systems augment sensitivity of MDR pathogens to antibiotics by circumventing resistance mechanisms such as impenetrability to antibiotics due to modification of the outer membrane porin proteins since the drug delivery system-antibiotic complex presents changed conformation compared with the non-encapsulated antibiotic^20^. Thus, isolation of chitosan from readily available resources with potential future applications as antimicrobials and chitosan-based drug delivery system to combat antimicrobial resistance is necessary.

Therefore, this study was aimed at comparing the physicochemical properties of chitosan extracted from banana weevils, Nile perch scales and edible mushrooms with Commercial chitosan (Sigma Aldrich). Furthermore, for application purposes, the antibacterial activity of locally isolated chitosan was assessed against carbapenem resistant *E. coli* and *K. pneumoniae*.

## Results

### Composition by dry weight of chitin and chitosan

The composition of dry weight chitin was determined by using the ratio of the starting dry weight of the raw material (10 g) and the obtained chitin dry weight after demineralization and deproteinization. The dry weight of chitin obtained was 11.8%, 9.9% and 39% for banana weevils (BW), mushrooms (MSR) and Nile perch scales (NS) respectively with *P* values > 0.05 indicating variability between chitin yield from each raw material. The percentage yield of chitosan from chitin ranged from 70.2 to 82% with *P* value > 0.05 between BW and MSR chitosan, *P* values < 0.05 among BW and NS chitosan; MSR and NS chitosan. The chitosan yield from commercial chitin was statistically comparable to that of NS and significantly different from the chitosan yield of BW and MSR, Table 1.

**Table 1:** Dry weight yield of chitin and chitosan isolated from locally available materials.

Raw material	Chitin mean dry weight (%)	Chitosan mean dry weight (%)
Banana weevils	1.18 ± 0.02/11.8^A^	0.83 ± 0.02/70.2^A^
Mushroom	0.99 ± 0.02/9.9^B^	0.74 ± 0.03/74.0^A^
Nile perch scales	3.9 ± 0.42/39^C^	3.2 ± 0.32/82.1^B^
Commercial chitosan	N/A	0.85 ± 0.03/85.0^B^

Mean values in each column accompanied by the same letter are not significantly different (*P* > 0.05) (Tukey Multiple Comparison) and values accompanied by letter (s) which are not similar are significantly different (*P* < 0.05).

### Ash content and moisture content

In general, Chitin and chitosan from each raw material registered statistically similar ash contents. However, chitin and chitosan samples from MSR had the lowest ash content statistically similar to the ash content of commercial chitosan but significantly different (*P* values < 0.05) from the BW and NS chitin and chitosan ash content, Table 2. The moisture content varied from 3.5 to 6.4%, with *P* values > 0.05 for MSR and NS chitosan signifying analogous moisture content but < 0.05 for BW and commercial chitosan indicating considerable moisture content difference between BW and commercial chitosan and the other chitosan obtained from different locally available materials, Table 2.

**Table 2:** Physiochemical properties of chitin and chitosan isolated from locally available materials.

Raw materials	Chitin Ash content mean weight (g)/ percentage (%)	Chitosan ash content mean weight (g)/ Percentage (%)	Chitosan moisture content mean weight (g)/ Percentage (%)	Mean dry soluble weight (g)/ Solubility (%)	Chitosan residue ash content (%)	Chitosan residue N content (%)	Chitin N content (%)	Chitosan N content (%)	Chitosan MW (KDa)	DD (%)	CrI (%)
BW	0.024 ± 0.003/2.4^A^	0.022 ± 0.001/2.2^A^	0.064 ± 0.004/6.4^A^	0.84 ± 0.003/84^A^	81.1 ± 1.23^AB^	14.8 ± 0.50^A^	7.0 ± 1.23^A^	6.9 ± 0.32^A^	343 ± 37.3^A^	77.8 ± 0.39^A^	41.1 ± 0.52^A^
MSR	0.017 ± 0.002/1.7^B^	0.015 ± 0.001/1.55^B^	0.039 ± 0.001/3.9^B^	0.86 ± 0.003/86^A^	93.8 ± 1.23^B^	5.9 ± 0.19^B^	6.8 ± 0.37^A^	6.6 ± 0.19^A^	348 ± 35.0^A^	78.1 ± 0.54^A^	48.4 ± 0.44^B^
NS	0.035 ± 0.003/3.5^C^	0.035 ± 0.003/3.5^C^	0.035 ± 0.003/3.5^B^	0.69 ± 0.004/69^B^	63.0 ± 1.87^c^	30.8 ± 0.51^C^	8.2 ± 0.19^AB^	7.0 ± 0.55^AB^	291 ± 6.3^A^	79.1 ± 0.76^A^	51.1 ± 0.47^B^
C	0.015 ± 0.002/1.5^A^	0.015 ± 0.001/1.5^B^	0.067 ± 0.004/6.7^A^	0.87 ± 0.003/87^A^	93.1 ± 2.12^B^	5.7 ± 0.19^B^	6.8 ± 0.37^A^	6.7 ± 0.31^A^	361 ± 31.9^A^	76.0 ± 0.29^A^	59.6 ± 0.88^C^

Mean values in each column accompanied by the same letter are not significantly different (*P* > 0.05) (Tukey Multiple Comparison) and values accompanied by letter (s) which are not similar are significantly different (*P* < 0.05), N represents Nitrogen, g: gram, BW: Banana weevil, MSR: Mushroom, NS: Nile perch scale, C: Commercial, MW: Molecular Weight, KDa: Kilo Dalton, DD: Degree of Deacetylation and CrI: Crystalline Index.

### Nitrogen content

Chitosan samples registered lower nitrogen contents than chitin but statistically similar. The nitrogen content ranged from 6.8% in MSR chitin to 8.2% in NS chitin and 6.6% in MSR chitosan to 7.0 in NS chitosan. Nile perch scale chitin and chitosan had the highest nitrogen content but statistically comparable to the nitrogen content in BW, MSR and commercial chitin and chitosan, Table 2.

### Solubility

Chitosan isolated from locally available materials exhibited moderate solubility ranging from 69 to 86%, Table 2. Tukey comparison registered a *P* value > 0.05 among MSR, BW and commercial chitosan while a *P* value < 0.05 was recorded between MSR and NS chitosan, BW and NS chitosan. Furthermore, the residue after solubilizing chitosan was analyzed for ash and nitrogen contents. Nile perch scale chitosan residues yielded the lowest ash content and the highest nitrogen content substantially different from the ash content and nitrogen content values obtained from BW, MSR and commercial chitosan residues, Table 2.

### Molecular weight

Viscosity average molar mass/molecular weight (M_v)_ of the different chitosan samples was determined using intrinsic viscosity. The average molecular weight ranged from 291 KDa for NS chitosan to 348 KDa for MSR chitosan. Tukey multiple comparison generated *P* values > 0.05 showing that the molecular weights of chitosan isolated from different locally available materials are statistically similar and comparable with commercial chitosan.

### Infrared spectrophotometry determination of chitosan functional groups and DD

**Figure 1:**
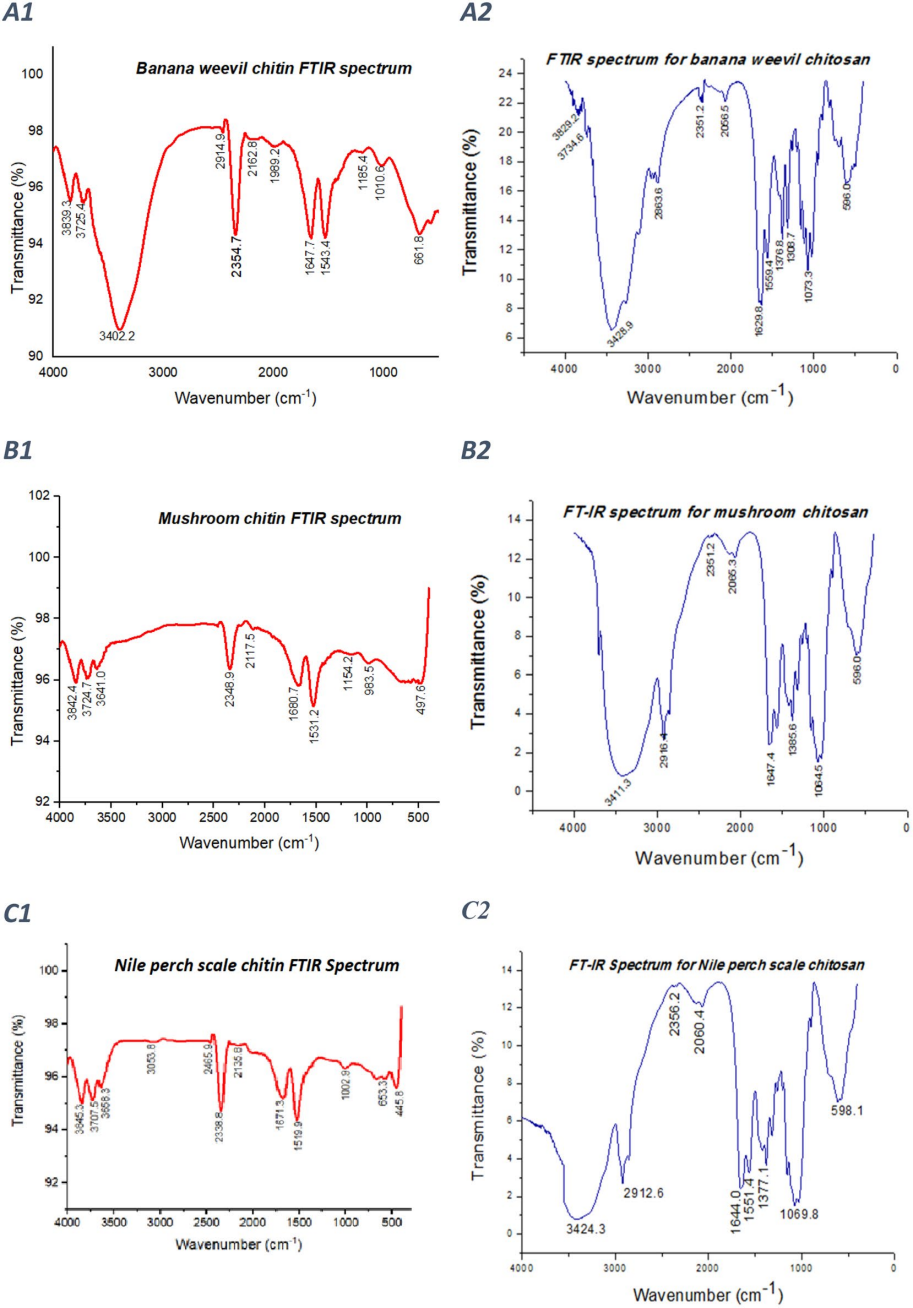

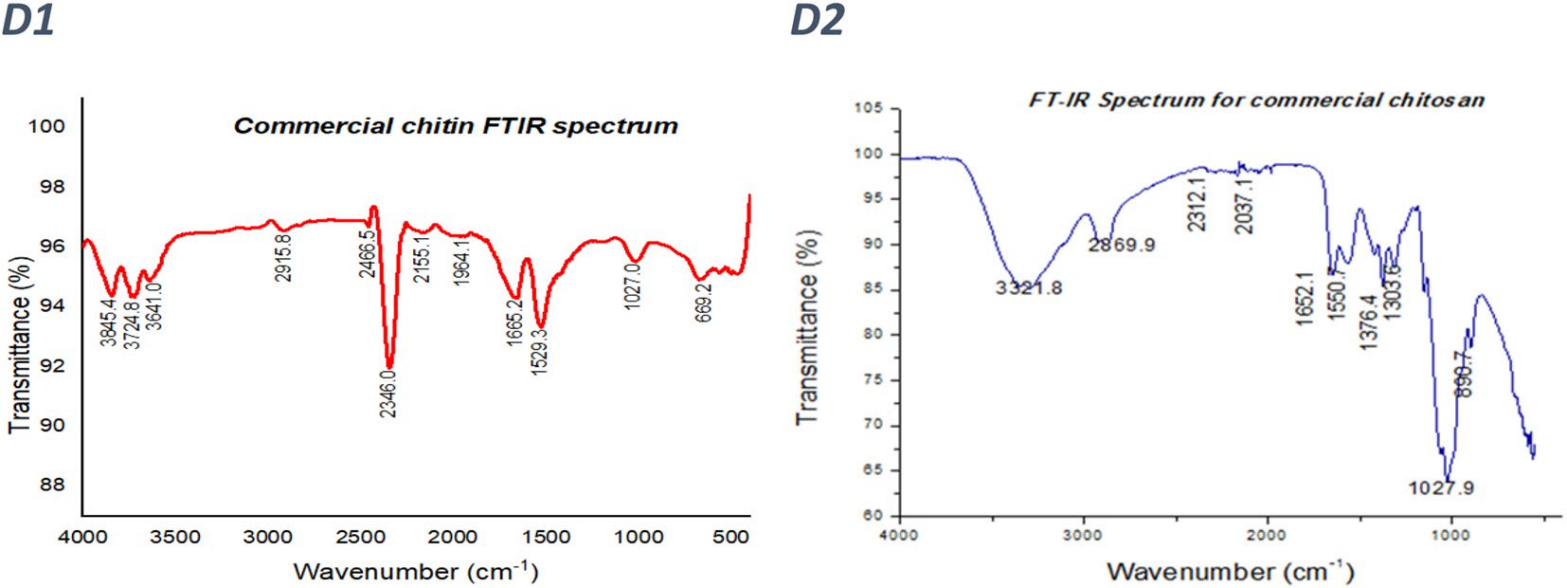
FTIR spectra for chitin and chitosan isolated from locally available materials; (**A1**) Banana weevil chitin FTIR spectrum, (**A2**) banana weevil chitosan FTIR spectrum, (**B1**) mushroom chitin FTIR spectrum, (**B2**) mushroom chitosan FTIR spectrum, (**C1**) Nile perch scale chitin FTIR spectrum, (**C2**) Nile perch scale chitin FTIR spectrum, (**D1**) commercial chitin FTIR spectrum, and (**D2**) commercial chitosan FTIR spectrum.

Fourier Transform Infrared spectroscopy of chitin and chitosan isolated from BW, MSR, NS and commercial chitosan (Sigma Aldrich) yielded spectra with functional groups shown in Table 3 and Fig. 1. For estimation of the DD of chitosan, bands 1629.8, 1647.4, 1640.8 and 1652.1 cm^−1^ for BW, MSR, NS and commercial chitosan respectively corresponding to acetylated residues of amide I (NHCOCH3) and 3428.5, 3411.3, 3448.7 and 3321.8 for BW, MSR, NS and commercial chitosan respectively associating to the vibration of the OH molecule were used^24^. Analysis by Infrared spectroscopy estimated the percentage DD as 77.8%, 78.1%, 79.1% for BW, MSR and NS chitosan respectively, Table 2.

**Table 3:** Comparison of FTIR characteristic bands for chitin and chitosan isolated from locally available materials and commercial chitosan band patterns.

Functional group range	Wavenumber (cm^−1^)								Functional group/Molecule
	Banana weevil		Mushroom		Nile perch scale		Commercial		
	Chitosan	Chitin	Chitosan	Chitin	Chitosan	Chitin	Chitosan	Chitin	
4000–3700	3829.2	3839.3	–	3842.4	–	3845.3	–	3845.4	O–H
4000–3700	3734.6	3725.4	–	3724.7	–	3707.5	–	3724.8	O–H
3650–3400	3428.9	3402.2	3411.3	3641.0	3424.3	3658.3	3321.8	3641.0	Group tension –OH
2919–2868	2863.6	2914.9	2916.4	–	2912.6	3053.8	2869.9	2915.8	Stretching band C–H and –C = O of the amide group CONH-R of the polymers
2349	2351.2	2354.7	2352.2	2348.9	2356.2	2465.9	2312.1	2466.5	Carbon dioxide O = C = O
2349	–	–	–	–	–	2338.8	–	2346.0	Carbon dioxide O = C = O
2140–1990	2056.5	2166.8	2065.3	2117.5	2060.4	2135.8	2037.1	2155.1	Isothiocyanate
2140–1990	–	1989.2	–	–	–	–	–	1964.1	Isothiocyanate
1650–1550	1629.8	1647.7	1647.4	1680.7	1644.0	1671.3	1652.1	–	Amide I
1560–1500	1559.4	1543.4	1544.0	1531.2	1551.4	1519.9	1550.7	1529.3	Doubling group NH_2_
1390–1370	1376.8	–	1385.6	–	1377.1	–	1376.4	–	Amide III
1310–1250	1308.7	1185.4	–	–	–	–	1303.6	1027.0	Aromatic band C–O
1124–1087	1073.3	1010.6	1064.5	1154.2	1069.8	1002.9	1027.9	–	Stretching band C–O–C
900–890	–	–	–	983.5	–	–	890.7	–	C—O—C bridge and glucosidic linkage of amides
800–600	596.0	661.8	596.0		598.1	653.3	–	669.2	Amide VI
	467.6	–	–	497.6	–	445.8	–	497.0	Amide VI

### XRD analysis

**Figure 2:**
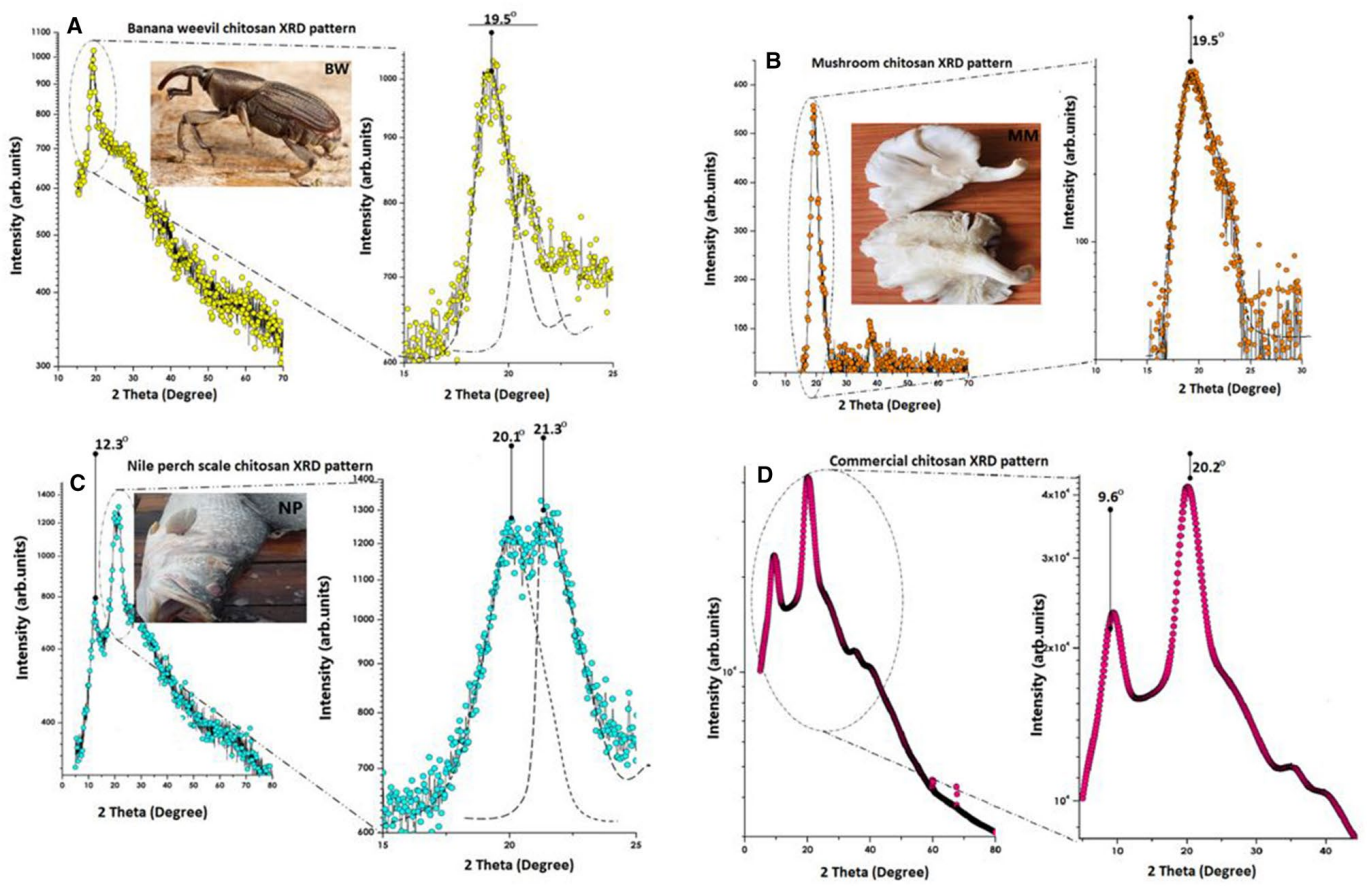
XRD patterns for chitosan isolated from locally available materials; (**A**) XRD pattern for banana weevil chitosan, (**B**) XRD pattern for mushroom chitosan, (**C**) XRD pattern for Nile perch scale chitosan, and (**D**) XRD pattern for commercial chitosan. Banana weevil (BW), mushroom (MM) and Nile perch (NP) pictures merged in XRD patterns (**A**), (**B**) and (**C**) were taken by author KS.

**Figure 3:**
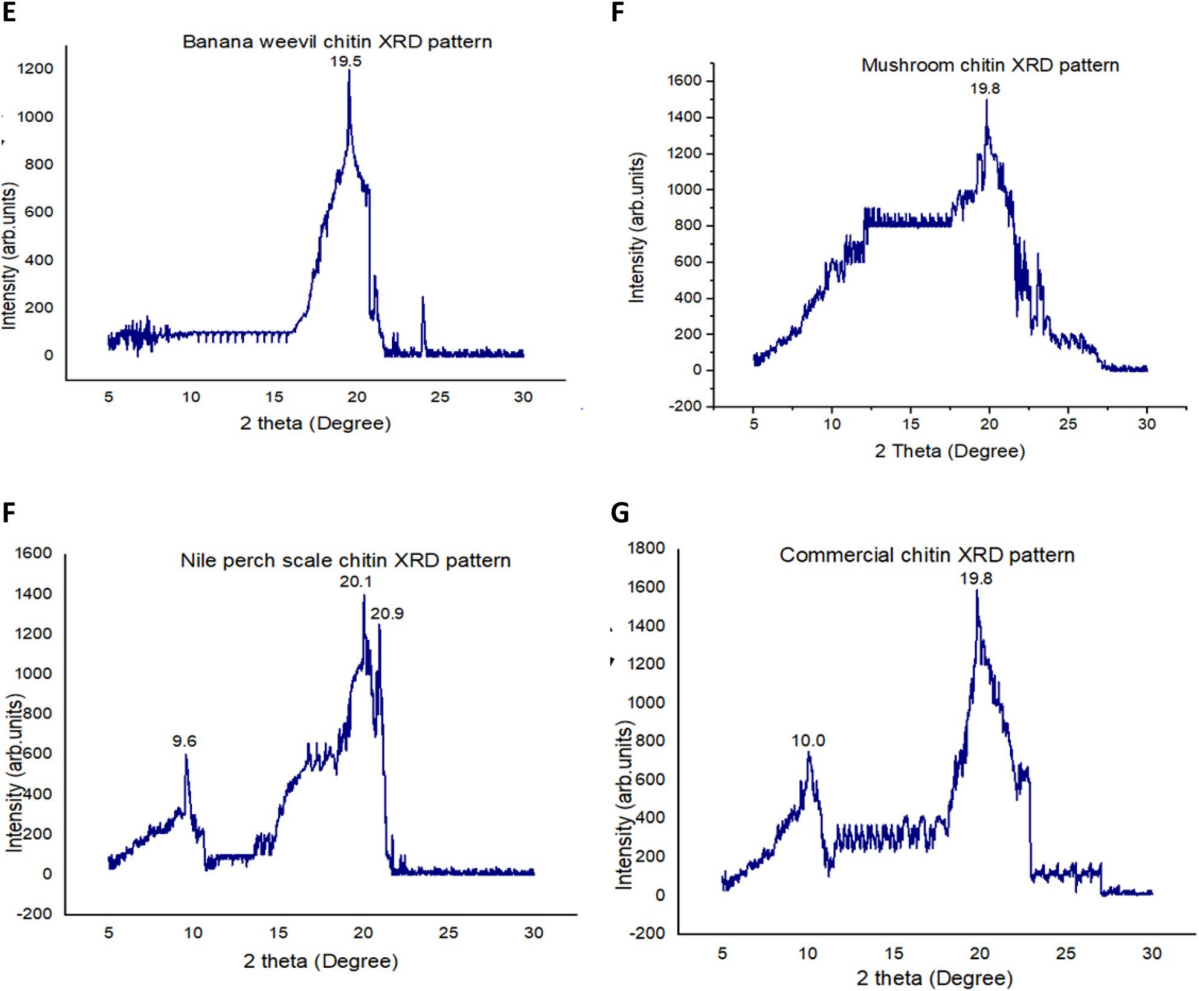
XRD patterns for chitin extracted from locally available material; (**E**) XRD pattern for banana weevil chitin, (**F**) XRD pattern for mushroom chitin, (**G**) XRD pattern for Nile perch scale chitin, and (**H**) XRD pattern for commercial chitin.

XRD patterns exhibited peaks at 2θ values of 19.5° for both chitosan extracted from BW (corresponding to different reticular plans with lattice periodicities of 4.570 Å and 4.530 Å) and MSR (corresponding to 4.407 Å and 4.136 Å reticular plans), Fig. 2A,B. Chitosan from NS registered 3 peaks at 2θ values of 12.3°, 20.1° and 21.3° with periodic reticular atomic plans lattice periodicities of 2.350 Å and 5.581 Å respectively, Fig. 2C, whereas commercial chitosan (control) scored 2 peaks at 2θ values of 9.6° and 20.2° with intereticular atomic periodicities of 4.414 Å and 9.201 Å, Fig. 2D. However, the peak intensities of chitosan isolated from locally available materials were lower than those attained by the control (23,061 a.u and 41,664 a.u), Fig. 2. Furthermore, XRD patterns exhibited by chitin are comparable to the XRD pattern of chitosan but with high intensity pointed peaks, Fig. 3. CrI calculated using XRD pattern ranged from 41% to 51.1% for BW and NS chitosan and Tukey multiple comparison revealed that the CrI for chitosan obtained from locally available materials was substantially different from that of commercial chitosan, Table 2.

### Bactericidal activity of chitosan

Chitosan isolated from locally available materials and commercial chitosan revealed concentration dependent antibacterial activity against carbapenem resistant and sensitive bacteria. No antibacterial activity was registered at all concentrations below 3000 µg/ml. Chitosan exhibited bactericidal activity when the concentration was increased above 3000 µg/ml. The mean inhibition zones increased with increasing chitosan concentration. MSR chitosan exhibited potent antibacterial activity with mean growth inhibition zones of 7 mm (3 mg/ml) and 11 mm (4 mg/ml) similar to those of commercial chitosan. BW chitosan followed with mean growth inhibition zones of 5 mm (3 mg/ml) and 7 mm (4 mg/ml). NS chitosan recorded the least antibacterial activity with mean growth suppression zones of 2 mm (3 mg/ml) and 4 mm (4 mg/ml), Table 4.

**Table 4:** Average growth inhibition zones of chitosan isolated from different sources. Meropenem (MEM) was used as a positive control while 1% acetic acid as a negative control.

Bacteria type	Zone of growth inhibition (mm)													
	MEM disk	1% acetic acid	Commercial chitosan (mg/ml)			BW (mg/ml)			MSR (mg/ml)			NS (mg/ml)		
			0.25–2	3	4	0.25–2	3	4	0.25–2	3	4	0.25–2	3	4
Carbapenem sensitive *E. coli*	40	0	0	7^A^	11^B^	0	5^A^	7^A^	0	7^A^	10.5^A^	0	2^C^	3^C^
Carbapenem resistant *E. coli*	0	0	0	7^A^	11^B^	0	5^A^	6.5^A^	0	8^A^	11^A^	0	2^C^	4^C^
Carbapenem sensitive *K. pneumoniae*	40	0	0	7^A^	11^B^	0	5^A^	6^A^	0	7^A^	11^A^	0	2^C^	4^C^
Carbapenem resistant *K. pneumoniae*	0	0	0	7^A^	11^B^	0	6^A^	6.5^A^	0	7^A^	11^A^	0	2^C^	4^C^

Mean values in each column accompanied by the same letter are not significantly different (*P* > 0.05) (Tukey Multiple Comparison) and values accompanied by letter (s) which are not similar are significantly different (*P* < 0.05).

## Discussion

The percentage of chitin obtained in previous studies ranged from 2.5 to 12.2% for insects^21^, 7.9–11.4% for mushrooms^22^ and 33–45% in fish scales^23^. The dry weight of chitin obtained in this study was 11.8%, 9.9% and 39% for banana weevils (BW), mushrooms (MSR) and Nile perch scales (NS) respectively falling within the ranges of the previous studies. Furthermore, the percentage yield of chitosan from chitin in this study ranged from 70.2 to 82%. These results corroborate well with the chitosan yield reported by Erdogan et al.^22^.

The residue that remains after complete pyrolysis of the material in the presence of air is termed as ash and is inorganic in nature^24^. Therefore, chitin and chitosan ash contents were determined gravimetrically and the ratio of chitosan weight burnt to the weight of inorganic residue was computed into percentages. Determination of ash content in chitin and chitosan is a vital litmus to assess the effectiveness of the demineralization process. The remnant minerals may include toxic inorganic elements such as Cadmium, Lead and Mercury that could pose health risks if such chitosan is used for biomedical applications as they are extremely hazardous at even very low levels of exposure^25–27^. Furthermore, solubility of chitosan is greatly affected by the presence of inorganic minerals as this subsequently lowers viscosity^24^. This greatly affects fabrication of chitosan-based drug delivery systems. Furthermore, the level of demineralization and deproteination determines the purity of chitosan which in turn affects its biological properties like immunogenicity, biocompatibility and biodegradability^28^. Chitosan with an ash content lower than 1% possesses superior biological properties and is recommended for biomedical applications^29^. Contrarily, several studies have used chitosan with ash content higher than 1% for biological applications^30–32^. Thus, chitosan generated by this study with ash contents ranging from 1.5 to 3% is fit for medical use. However, the demineralization step needs to be improved to reduce the ash content further to meet the regulatory requirements if the chitosan isolated from locally available materials is to be used in medical applications.

Chitosan has a great capacity to form hydrogen bonds with water through both its hydroxyl and amino groups hence its hygroscopic in nature. The quantity of adsorbed moisture relies on the initial moisture content of the raw materials and storage environmental conditions^33^. The moisture value of commercial chitosan powder ranges from 7 to 11% (w/w) and not influenced by degree of deacetylation or molecular weight^34^. Moisture content is one of the most important factors which influence the usability of chitosan powder during drug carrier and tablet preparations. Moisture content level should be put into consideration when formulating chitosan-based drugs to reduce pharmaceutical powder faults especially after storage as water content above 6% affects powder flow properties, compressibility and tensile strength of the tablets^28^. The moisture content of the chitosan isolated from locally available material was within the recommended range hence suitable for pharmaceutical use.

Chitosan isolated from locally available materials exhibited moderate solubility ranging from 69 to 86%. Contrary to this, Nessa et al.^29^ reported excellent solubility ranging from 96.0 to 97.2% of chitosan isolated in-house from prawn shells. Low to moderate solubility values of chitosan are attributed to high protein content and low DD^35^. Nevertheless, this study achieved high DD comparable to levels reported by other studies and commercial chitosan. Therefore, this moderate solubility may be attributed to low demineralization^35^ and distribution of the remaining acetyl groups (glucosamine and N-acetylglucosamine units) along the polymer chain which is termed as the pattern of deacetylation (PA)^36^. PA substantially impacts on the charge density which in turn influences the solubility of chitosan regardless of the DD and molecular weight^37^. Indeed, analysis of the residues that remained after solubilizing chitosan in 1% acetic acid revealed that the residues mainly contained inorganic materials with ash contents of over 81.1% for BW, MSR and commercial chitosan and the remaining percentages majorly contributed by proteins as depicted by the nitrogen content^38^. Furthermore, this study revealed nitrogen content of 7.0%, 6.8%, 8.2% and 6.8% for BW, MSR, NS and commercial chitins respectively. Statistically similar nitrogen contents were registered by the respective chitosans. The nitrogen content of chitin and chitosan is extremely a vital measure of purity. The nitrogen level of fully acetylated chitin is 6.89%^39,40^. Nitrogen values greater than 6.89% suggest presence of proteins hence low level of deproteination, whereas nitrogen content below 6.89% postulates ineffective demineralization step^41^. This explains why the insoluble chitosan residues had considerably higher ash content and to some extent nitrogen bearing compounds. High mineral and residual proteins contents may cause complications in chitosan dissolution and hinder designing and development of chitosan matrix-based drug delivery systems. Total elimination of minerals and proteins during chitosan isolation is impractical as the process requires use of extremely concentrated acids and bases respectively at higher temperatures which yields degraded chitosan. However, increasing the duration of the demineralization and deproteination steps only possibly may farther decrease the ash and protein contents to the recommended levels.

Molecular weight is one of the most important physiochemical properties that influences other physicochemical and biological behaviors such as hydrophilicity, viscosity, moisture absorption, biodegradability, antimicrobial activity and mucoadhesion of chitosan^6^. With respect to the raw material and isolation method, the molecular weight of commercial chitosan ranges from 10 KDa to 100,000 kDa. For example, the process of deacetylation may lower the molecular weight of the polymer^28^. Pharmaceutical industries have widely exploited chitosan in several forms of drug delivery systems such as tablets, nanocarriers, hydrogels microspheres, micelles among others. Due to the high viscosity, high molecular weight chitosan based drug carriers discharge the active ingredient gradually and in a controlled manner prolonging the duration of drug activity hence improving treatment outcomes as well as decreasing the drug side effects^42^. In contrast, low molecular weight chitosan possesses high penetrative power than high molecular weight chitosan, thus, can effectively infiltrate bacterial cell walls, bind DNA, block the process of transcription hence inhibit the protein synthesis. Thus, low molecular weight chitosan possesses potent antimicrobial activity^43,44^. On the other hand, high molecular weight chitosan at higher concentrations can exhibit antibacterial activity through binding to the negatively charged bacterial cell wall parts through electrostatic interactions forming an impermeable coating around the cell, hence blocking movement of materials into and out of the cell. Thus, the molecular weight of chitosan should be determined to ensure that it meets the quality for biomedical application. In this study, a relatively high molecular weight chitosan varying between 291 and 348 KDa was obtained. This fairly high molecular weight chitosan isolated from locally available materials falls within the range of chitosan recommended for designing and development of drug delivery system.

Fourier Transform Infrared spectrophotometric examination of chitosan isolated from BW, MSR, NS and commercial chitosan (Sigma Aldrich) yielded comparable spectra an indication that locally isolated chitosan has similar physicochemical properties due to the presence of almost similar functional groups. Similar FTIR results were obtained for chitin. However, chitin samples were more hydrated than their respective chitosan as shown by presence of extra 2 bands signifying OH groups between 4000 and 3700 cm^−1^. Chitin from various sources is mainly grouped into α and β polymorphs and rarely the γ type. Chitin is made of fibres that are arranged in layers. In α–chitin the adjacent chains are arranged in opposite directions and in an anti-parallel arrangement whereas in β–chitin, the adjacent layers are in the same direction and parallel. In γ–chitin, every 3rd layer is in the opposite direction as compared to the two precedent layers^45^. Beta chitin display bands for CH_X_ deformation at a wavelength of approximately 1455 cm^−1^ and 1374 cm^−1^ and several narrow peaks in the C–O–C and C–O stretching region of 1200–950 cm^−1^ not present in α chitin^46^. Comparable FTIR spectra were observed in this study indicating presence of α chitin in BW, MSR and NS. The percentage chitosan DD varied from77.8 to 79.1%. These DD values are consistent with the shrimp commercial chitosan DD value (76%) used as a control in this study and DD values reported by other studies. Liu et al.^47^ and Santos et al.^48^ reported DD values of 73.1% and 76% respectively. Furthermore, the DD values obtained in this study are within the range of 75 and 90% deacetylation degree in industrial processing^49^. One of the most important factors that should be considered when isolating chitosan in-house for biomedical application is DD. Degree of deacetylation influences several chitosan traits that include biological, physicochemical and mechanical properties. It was reported that chitosan polymer with low DD disintegrated fast and induced an acute inflammatory response while highly deacetylated chitosan induced negligible inflammation hence biocompatible^50^. Furthermore, swelling property is one of the most vital factors that impact the chitosan performance in the biomedical field. High level of swelling reduces the elasticity and tensile strength of chitosan pharmaceutical materials which increases the risk of collapsing. Chitosan with low DD has a higher swelling index. When water molecules are absorbed and combine with the polar groups in the material molecules, the material swells. Pharmaceutical materials designed from chitosan with higher DD exhibited lower swelling index an indication that highly deacetylated chitosans are suitable for clinical applications^51^. Additionally, chitosan chains with higher DD are more flexible and flexible chains will enhance the formation of hydrogen bonds, boosting the tensile strength and elasticity of chitosan material as a whole^51,52^. Thus, the DD influences the mechanical property of chitosan. Preparation of chitosan therapeutic formulations involves use of solvents and the mechanical strength is compromised by absorption of water by the hydrophilic regions of chitosan. However, this can be overcome by use of chitosan with higher DD. Thus, cautious isolation and purification of chitosan with appropriate DD specifically for fabrication of chitosan-based formulations for parenteral biomedical application should be of great interest.

The 2θ values obtained from chitosan isolated from locally available materials were within the same range with the XRD patterns (2θ angles 9.6° and 20.2°) registered by commercial chitosan (control) used in this study. Furthermore, peaks at 2θ = 10° and 2θ = 20° of commercial chitosan (Sigma Aldrich) have been exhibited by X-ray diffraction studies^53^. The results from this study are comparable to the established chitosan XRD pattern as peaks at 2θ values of 12.3°, 20.1° and 21.3° for Nile perch scales chitosan, 19.5° for banana weevil and mushroom chitosan were registered. In banana weevil and mushroom chitosan, the weak peak at 2θ = 10° disappeared. Similar deviations were registered by other studies which attempted to extract chitosan from locally available materials^53–56^. However, high peak intensity for Nile perch scale chitosan and a slight shift in 2θ = 20° diffractive angle for all extracted chitosan indicates that this study achieved highly crystalline chitosan. The XRD patterns of commercial chitin and that isolated from different locally available are analogous to those of their respective chitosan. However, a slight variation exists in the intensity of peaks. Chitin XRD pattern displayed higher intensity of peaks than chitosan. Similar results were observed in NS chitosan XRD spectrum. High intensity peaks in NS chitosan and chitin is mainly as a result of high concentration of impurities such as minerals and proteins. Furthermore, XRD analysis showed only peaks at 2θ values associated with hydrated polymorphs for all the chitosans isolated from locally available materials and the commercial chitosan. This is in agreement with the XRD spectrum of the hydrated chitosan obtained by other studies^57,58^. The CrI values were obtained by Focher et al.^59^ methods using formula (9). However, the CrI estimated by this method might be too low as the procedure has been implicated to underestimate CrI, because of overestimation of the input of the amorphous phase^57,60^. Indeed, this study achieved low CrI values but higher than the CrI values reported by De Queiroz Antonino et al.^60^ who used Osorio-Madrazo et al.^57^ improved method.

A substantial variation was observed in the yield, ash content, moisture content, solubility and crystallinity of chitosan isolated from locally available materials. The variation in physicochemical properties among chitosan obtained from BW, MSR and NS is in line with other studies. Szymańska and Winnicka^28^ observed that a variety of chitosan raw materials lead to considerable dissimilarities in the quality and properties of chitosan and its products. Thus, significant deviations from the pharmacopeial recommendation might be registered by chitosan obtained from different sources.

Recently, substantial research attempts have been made to investigate the antimicrobial activity of chitosan. It has been reported that chitosan possesses potent antibacterial and antifungal activity^61–65^. Vilar Junior et al.^61^ reported that chitosan exhibited minimum inhibitory concentration ranging from 78 to 625 µg/ml in in vitro studies. However, the antibacterial activity of chitosan isolated from locally available materials and commercial chitosan in this study was attained at very high concentrations of 3000 µg/ml and 4000 µg/ml. Kamjumphol et al.^66^ reported similar results where chitosan antibacterial activity was dose dependent and the most efficacious concentration was 5000 µg/ml. The low antibacterial activity may be attributed to fairly high molecular weight of chitosan. In general, the antimicrobial activity of chitosan against *E. coli* increases with increasing molecular weight but up to a certain level. Tanigawa et al.^67^ observed that Chitosan of 80 KDa exhibited superior antimicrobial activity against *E. coli* as compared to 166 KDa, 190 KDa and very low molecular weight chitosan of 2 to 12 KDa. Similar results were reported by several studies^68–70^. Indeed, this study isolated fairly high molecular weight chitosan. Furthermore, low antibacterial activity of chitosan in this study may also be associated to the average DD as antibacterial activity of chitosan increases with increase in the DD. Chitosan with high DD of over 90% possesses a higher positive charge density that facilitates electrostatic interaction with the negatively charged bacterial cell thereby conferring more potent bactericidal activity than chitosan with moderate DD^71,72^. Additionally, the dose dependent antibacterial activity achieved in this study may be due to increase in the net positive charge as the concentration of chitosan increases. Moreover, the low antibacterial activity in this study may due to high ash content above the recommended value (1%) which affects the physicochemical properties of chitosan such as solubility which in turn negatively affects its bioavailability. Indeed, low solubility affects the bioavailability of chitosan. NS chitosan with the lowest solubility exhibited the least antibacterial activity.

## Conclusion

The purity level of chitosan and its physicochemical properties affect its biological parameters such as biodegradability, biocompatibility and antimicrobial activity. These physicochemical characteristics are influenced by raw materials and the method used in chitosan isolation. Most literature documented shrimp shells and other crustaceans as the main raw materials for high grade chitosan. Basing on this background, this study isolated chitosan from banana weevils, mushrooms and Nile perch scales. Chitosan isolated from the locally available materials exhibited moderately high DD and other physicochemical properties corroborating with commercial chitosan (Sigma Aldrich) but with moderate solubility and antibacterial activity. Therefore, attempts should be made to improve the chitosan isolation methods so that the DD and solubility are further increased while the inorganic and protein contaminants are completely eliminated. This should result into optimal chitosan isolation suitable for pharmaceutical and biomedical applications. Furthermore, the cell membrane of bacterial cells is negatively charged. Thus, the zeta potential of chitosan intended for antibacterial application should be determined as only positively charged materials with a pH lower than 6.5 interact with the negatively charged components of the bacterial cell wall.

## Methods and materials

### Source of materials

Nile perch scale wastes were obtained from a local fish market while banana weevils and edible mushroom were collected from National Agricultural Research Laboratories, Kawanda. Shrimp shell chitosan (CAS number: 9012-76-4; 448877-50G) and chitin (CAS number: 1398-61-4; C7170-100G) were purchased from Sigma Aldrich. Carbapenem resistant *E. coli* and *K. pneumoniae* were a kind donation from Department of Microbiology, College of Health Sciences, Makerere University.

### Study design and site

This was a laboratory-based study conducted from College of Veterinary Medicine Animal Resources and Biosecurity, Makerere University, iThemba LABs, Cape Town and University of South Africa (UNISA). Isolation of chitin and chitosan was performed from the Pharmacology Laboratory, Makerere University, while chitosan antibacterial activity was evaluated from the Central Diagnostic Laboratory, Makerere University. Characterization of chitin and chitosan was conducted from iThemba LABs and UNISA. Commercial shrimp shell chitin and chitosan were used as controls in all characterization experiments.

### Chitin extraction

Banana weevils, Nile perch scales and mushrooms were cleaned using running tap water and finally rinsed in distilled water. The cleaned weevils, scales and mushrooms were oven dried at 60 °C for 1 week and then ground to powder using an electric miller. Chitin was extracted from the resultant powder following Mohammed et al.^73^ adjusted procedure. Demineralization was carried out by treatment of the banana weevil, Mushroom and Nile perch scale powders with 1.0 M HCl solution at 50 °C in a water bath for 24 h with a solution to solid ratio of 15 mL/g. This step was replicated ten times. The mixture was centrifuged at a speed of 4000 × g for 10 min using Thermo Scientific™ Fiberlite™F6-10 × 1000 LEX roto centrifuge. The resultant sediment was washed with distilled deionized water until neutral pH was achieved. The sediment was deproteinized by adding 1.0 M sodium hydroxide at a ratio of 15 mL:1 g and then heated at 80 °C for 8 h in a water bath. This treatment was repeated four times. The resultant chitin was then washed with distilled deionized water to neutrality. Finally, chitin was washed by boiling in hot absolute ethanol and later in absolute acetone in a water bath for 10 min to remove any impurities. The purified chitin was dried in a vacuum oven at 50 °C to constant weight. The chitin content was determined by computing the weight differences between the raw materials and that of the chitin obtained after acid and alkaline treatments.

### Chitosan preparation

Chitin was treated with 50% NaOH (15 mL/g) at 90 °C in a water bath for 10 h with continuous mixing using a magnetic stirrer after which the resultant mixture was centrifuged at 4000 × *g* for 10 min using a Thermo Scientific™ Fiberlite™F6-10 × 1000 LEX roto centrifuge. The residue was washed with hot distilled deionized water until neutrality. The obtained chitosan was dried in a vacuum oven at 40 °C for 48 h. All the chitosan samples were purified by dissolving in 1% acetic acid and reprecipitated in 20% NaOH solution followed by centrifugation at 6000 × *g* for 10 min using a Thermo Scientific™ Fiberlite™F6-10 × 1000 LEX roto centrifuge to sediment chitosan. The sedimented chitosan was washed with distilled deionized water until a neutral pH, lyophilized and stored at − 20 °C until further use. The percentage chitosan yield was computed as a fraction of weight of dry chitosan and dry chitin from which it was generated.

### Characterization of chitin and chitosan

#### Estimation of the ash content of chitin and chitosan

The ash content of each chitin and chitosan sample was gravimetrically estimated after the pyrolysis of 1 g in a muffle furnace at 650 °C for 5 h. This procedure was done in triplicates and the mean ash content computed. The ash content was computed as a fraction of mass of the residue (MR) and mass of the sample (MS) using the formula (1) that follows;

1 $${\text{Percentage ash content}} = \frac{MR }{{MS}} \times 100;$$

where MS and MR are the weights (in grams) of the initial sample of the sample and residue respectively^74^.

#### Nitrogen content of chitin and chitosan

Amino acids are building blocks of proteins and contain nitrogen. Thus, nitrogen content is representative of protein content as the percentage of nitrogen present in a sample is directly proportional to the percentage of proteins. It is estimated that 1 g of a given protein sample contains 0.16 g of nitrogen. However, this value varies greatly depending on the protein source^75,76^. Therefore, nitrogen content can be used to infer the amount of protein in a sample. Nitrogen content was estimated by the Kjeldahl method^77,78^. Briefly, 1 g of each chitin and chitosan samples was hydrolyzed at 420 °C for 2 h in 15 ml of concentrated sulphuric acid (98% W/W) holding two copper catalyst tablets using a DT 220 digestor™, heat block (FOSS analytical, Denmark). After cooling, distilled deionized water (60 ml) was added to the hydrolysate followed by 50 ml of 60% NaOH to liberate ammonia, then distillation to recover the ammonia in 4% boric acid receiver. To quantify the amount of ammonia trapped, the receiving solution was titrated with 0.1 M HCl and the amount of nitrogen calculated using the formula (2) below;

2 $$1.0\,{\text{ml}}\,0.010\,{\text{N}}\,{\text{HCl}} = 10\,\upmu {\text{Mol}}\,{\text{N}}$$

#### Moisture content of chitin and chitosan

The water content of chitin and chitosan samples was assessed by gravimetric technique. This method involved drying of the samples until a constant mass in a vacuum oven at 105 °C for 24 h. This experiment was done thrice and the average moisture content was calculated. The water content was computed as the difference between the wet weight (WW) and dry weight (DW) of samples per gram using the formula (3) that follows:

3 $${\text{Moisture content percentage}} = \left( {\frac{WW - DW }{{{\text{WW}}}}} \right) \times 100;$$

where *WW* is the wet weight of samples and *DW* is the dry weight of samples after oven drying^16^.

#### Determination of chitosan solubility

A 1% solution of chitosan was constituted by adding 0.1 g (W1) of each chitosan sample previously dried at 105 °C for 24 h into 10 ml of 1% acetic acid in 15 ml falcon tube. The tubes were sealed and placed in an overhead shaker running at 60 rpm for 48 h. The solution was centrifuged at10,000 × g for 15 min using a Thermo Scientific™ Fiberlite™F6-10 × 1000 LEX roto centrifuge. The liquid phase was poured off and the sedimented residue was washed with 10 ml of distilled deionized water and centrifuged at 10,000 rpm for 15 min. The supernatant was decanted and the residue dried at 105 °C for 24 h (W2). This experiment was done three times and mean dry residue calculated. The dry residue was weighed and the percentage of solubility was determined using the formula (4) that follows;

4 $${\text{Percentage solubility}} = \frac{{\left( {W1 - W2} \right)}}{W1} \times 100;$$

where; *W1* was the initial weight of dry chitosan and *W2* was the weight of the dried residue.

Furthermore, the ash and nitrogen contents of the residues were determined.

#### Chitosan molecular weight estimation

The molecular weight of chitosan was determined using the intrinsic viscosity (ƞ) following Costa et al.^79^ adjusted method. A solvent medium was constituted by mixing 0.25 M acetic acid and 0.25 M sodium acetate at 1:1 ratio. Five hundred milligram (500 mg) of each chitosan sample was dissolved in 100 ml of the solvent medium to attain a chitosan concentration of 0.005 g/ml. The mixtures were left to stand for 24 h under constant stirring for complete solubilization of chitosan. Intrinsic viscosity was estimated using an automated Ubbelohde-type glass capillary with capillary tube diameter of 0.63 mm at 25 ± 01 °C. Determination of the intrinsic viscosity was achieved by recording the time of the solvents flow which included the flow of the solvent and the four chitosan solutions. This step was repeated three times to obtain the average flow rate for the solvent and the chitosan solutions. The rate of solvent flow was used to calculate intrinsic viscosities values by means of a single concentration value using Solomon and Ciuta^80^ Eq. (5). Obtained intrinsic viscosities were employed to estimate the molar mass or molecular weight of different chitosan samples using Mark–Houwink formula (6)^81^.

5 $$\left[ \eta \right]SC = \frac{{[2\left( {\eta sp - ln\eta r} \right)]^{0.5} }}{C}$$

where [ƞ]_SC_ is the intrinsic viscosity from Solomon and Ciuta equation, ƞ_r_ is the relative viscosity; (ƞ_r_ = t/t_0_ where t_0_ is the efflux time of the solvent and t is the efflux time of chitosan solution of a given concentration), ƞ_sp_ is specific viscosity (ƞ_sp_ = ƞ_r _– 1), ln is natural log and C is solvent concentration.

6 $$\left[ \eta \right] = {\text{k}}\left[ {{\text{M}}_{{\text{v}}} } \right]^{\upalpha }$$

where M_v_ is the viscosity average molecular weight of polymer, *α* and *k* are constants (*α* = 0*.*83 and *k* = 1*.*4 × 10^*−*4^ for 0.25 M acetic acid and 0.25 M sodium acetate solvent system^82^ and [*η*] is the intrinsic viscosity.

#### Fourier transform infra-red spectroscopy (FTIR)

Three milligrams (3 mg) of each sample (chitin and chitosan) and 5 g of Potassium bromide (KBr) were dried at 60 °C and 120 °C respectively under reduced pressure for 12 h. Each dried chitin and chitosan sample was homogenized with 100 mg of KBr and then compressed to form very thin discs of approximately 0.2 mm thickness. The chitin and chitosan samples were examined at 4000–400 cm^−1^ Wavenumber range using a PerkinElmer FT-IR Spectrometer. The spectrometer was set to perform at least 64 scans per sample. A KBr disc was used as reference. Functional group assigning to the generated FTIR spectra bands was done using documented literature^46,83–89^.

#### Determination of the degree of deacetylation (DD%)

The acetylation and deacetylation percentage of chitosan samples was determined by Fourier Transform Infrared Spectroscopy (FTIR). This was done through the correlation of some absorbance bands linked to some of amide, methyl and hydroxyl bands registered by the FTIR spectra. Vilar Junior et al.^61^ used the Amide-I band with a wavenumber of 1655 cm^−1^ and the hydroxyl group band at 3450 cm^−1^ using the formulae (7 and 8) that follow to determine the degree of acetylation (DA) and then the DD^90^;

7 $${\text{DA}}\left( \% \right) = \frac{A1655}{{3450}} \times \frac{100}{{1.33}}$$

8 $${\text{DD}}\left( \% \right) = 100 - {\text{DA}}$$

where A1655 was the absorbance at 1655 cm^−1^ of the Amide-I band which is measure of the N-acetyl group content, A3450 was the absorbance at 3450 cm^−1^ corresponding to the hydroxyl band as an internal standard to correct the disc thickness, factor 1.33 is the ratio of A1655 and A3450 for fully N-acetylated chitosan.

#### X-ray diffraction analysis (XRD)

X-ray diffraction was used to determine the crystallinity of the isolated chitin and chitosan where 500 mg of each sample chitosan powder were analyzed employing BRUKER AXS diffractometer, D8 Advance (Germany) fitted with Cu-Kα radiation (λKα1 = 1.5406 Å) from 2θ = 0.5° to 130°, with increment ∆2ϑ: (0.034°), voltage of 40 kV, current of 40 mA, power of 1.6 kW and counting time of 0.5 s/step. Generated data was analyzed by OriginPro Version 8.5 and resultant peaks 2θ values were compared with the commercial shrimp chitosan from Sigma Aldrich. The crystalline Index (CrI) values were determined from the XRD pattern following Focher et al.^59^ methods using formula 9.

9 $$Crl = \frac{{\left[ {\left( {I200 - Iam} \right)} \right]}}{I200} \times 100$$

where *I*_200_ is the maximum peak intensity for each chitosan at 2Ɵ–20° and *I*_am_ is the intensity of amorphous diffraction at 2Ɵ–16°.

#### Antimicrobial susceptibility assay of chitosan

Antibacterial activity of the chitosan was evaluated using standardized inocula of 1 × 10^7^ CFU/mL with 0.5 McFarland standards streaked onto the surface of sterile agar plates. Carbapenem resistant *E. coli* and *K. pneumoniae* suspended in Brain Heart Infusion Broth were inoculated onto the Mueller Hinton Agar plates and round wells of diameter 6 mm, depth 3 mm were prepared using a sterile cork borer in which 25 µl of chitosan solution (0.25, 0.5, 0.75, 1, 1.5, 2, 3 and 4 mg/ml in 1% acetic) were pipetted. Meropenem disks were used as the positive control while 1% acetic acid as negative control. The plates were incubated at 37 °C for 72 h. Zones of bacterial growth inhibition for each concentration were measured and record in millimeter (mm).

### Statistical analysis

Data analysis was done using Graph Pad Prism version 7.01. Comparisons of chitin yield, chitosan yield, ash content, moisture content, nitrogen content, solubility, molecular weight and DD among the chitin and chitosan samples isolated from BW, MSR and NS as well as commercial chitosan were performed using One-way analysis of variance (ANOVA) followed by Tukey's multiple comparisons test. Furthermore, comparison of mean zone (diameter in millimeter) of inhibition for each chitosan concentration was computed by one-way ANOVA. A *P* value of ≤ 0.05 indicated substantial statistical variance.

## Data Availability

All relevant data has been submitted with the manuscript and therefore no supplementary data.

## Abbreviations

2θ: Two theta
ANOVA: Analysis of variance
BW: Banana weevils
C-2: Carbon atom in position two
CFU: Colony forming unit
DA: Degree of acetylation
DD: Degree of deacetylation
FTIR: Fourier Transform Infrared Spectroscope
HCL: Hydrochloric acid
KBR: Potassium bromide
MSR: Mushroom
NaOH: Sodium hydroxide
NS: Nile perch scales
R-NHCOCH3: Acetyl residue
UNISA: University of South Africa
XRD: X-ray diffraction
